# High Hepatitis B Seroprevalence, Low Knowledge, and Poor Attitude towards Hepatitis B Virus Infection among Market Women in Bolgatanga Metropolis in the Upper East Region of Ghana

**DOI:** 10.1155/2020/4219413

**Published:** 2020-05-27

**Authors:** Gideon K. Helegbe, Faiza Tanko, Paul A. Aryee, Setor Aku Lotsu, Mathias J. A. Asaarik, Frank Anaba

**Affiliations:** ^1^Department of Biochemistry and Molecular Medicine, School of Medicine and Health Sciences, University for Development Sciences, Tamale, Ghana; ^2^Department of Public Health, School of Allied Health Sciences, University for Development Sciences, Tamale, Ghana; ^3^Department of Nutritional Sciences, School of Allied Health Sciences, University for Development Sciences, Tamale, Ghana; ^4^University Library, Nyankpala, University for Development Studies, Tamale, Ghana; ^5^Department of Public Health, Tamale Teaching Hospital, Tamale, Ghana

## Abstract

The Bolgatanga Municipal Health Directorate has reported liver cirrhosis among the first three diseases causing mortality from 2013 to 2015. This implicates hepatitis B virus (HBV) infection considering its high prevalence among blood donors in the Upper East Region of Ghana. However, for a vulnerable group such as market women, there is not much information with regard to the prevalence, knowledge, and attitude towards HBV infection. Thus, this study sought to bridge this gap by determining the seroprevalence, knowledge, and attitude of market women in the Bolgatanga Municipality of Ghana, towards HBV infection. A cross-sectional descriptive study was conducted (from October 2017 to March 2018) among 404 market women using a pretested questionnaire to ascertain the knowledge and attitudes of market women towards HBV infection, while hepatitis B surface Antigen Rapid Diagnostic Test strips were used to screen for the infection. The study revealed that the seroprevalence of hepatitis B among the market women was 15.6%, and majority of the study subjects (>60%) were unaware of HBV infection. Overall, knowledge on and attitude towards HBV infection were low and poor, respectively, with a significantly high number of the market women not wanting infected individuals to be isolated (*p*=0.049). A high seroprevalence, together with poor attitude and low knowledge levels, as seen in this study is of great public health concern. The study recommends regular HBV screening for market women for prompt treatment and vaccination as well as continuous health education to increase knowledge level and improve the poor attitudes of market women towards HBV infection.

## 1. Introduction

Viral hepatitis is a worldwide public health problem affecting over 500 million people globally, of whom almost 1 million die every year because of hepatitis infection. Opportunistic diseases like cirrhosis or liver cancer are common with hepatitis infection [1]. Of the five distinct viral hepatitis infections (A, B, C, D, and E), 95% hepatitis-related sickness and untimely deaths are caused by chronic hepatitis B and C infections (https://www.afro.who.int/health-topics/hepatitis). Chronic hepatitis affects over 70 million Africans (made up of 60 million with hepatitis B and 10 million with hepatitis C), with the youth being affected the most. This results in serious financial difficulties due to the high cost of treatment from the advanced liver disease and associated emotional distress and stigmatization (https://www.afro.who.int/health-topics/hepatitis).

Worldwide, the hepatitis B virus (HBV) is one of the most chronic infections with an estimated 257 million chronically infected people and a leading cause of hepatocellular carcinoma (HCC); indicatively, roughly 30% of the world's population show serological evidence of current or past infection [2]. It is also observed that the global burden of hepatitis B is severe with a projected number of 370 million people or more being chronic carriers [3]. Arguably, the sample size, study area, and the study population could have counted for the difference between the incidence reported in these studies [2, 3].

Most people who are infected with HBV or HCV are unaware of their chronic infection status and so can unknowingly transmit the infection to others. These people hardly go for medical checkups for the diagnosis to be known and would have transmitted to many people [4]. The likelihood of an HBV infection becoming chronic depends on the age at which the individual gets infected, with young people being the most likely to develop chronic infection. About 90% of infants infected during the first year of life and 30–50% of children infected between 1 and 4 years of age develop chronic infections, whilst about 25% of adults who become chronically infected during childhood die from HBV-related liver cancer or cirrhosis [1]. HBV diagnosis is usually confirmed by testing blood for parts of the virus and for antibodies against the virus [5].

In Africa and West Africa, the prevalence of hepatitis B is 10% and 15%, respectively [5]. In Ghana, HBV is considered to be of significant public health importance and its prevalence as detected by HBsAg seropositivity is between 10 and 12.3% (https://cdafound.org/dashboard/polaris/dashboard.html, [6]). Ghana Health Service reported in 2009 very precarious figures suggesting an increase in the prevalence ratio of viral hepatitis from 8 : 1 in 2005 to 6 : 1 in 2009 [7]. Despite many efforts towards the treatment and effective vaccination against hepatitis B, it is still considered the most dangerous type of viral hepatitis [8].

Reports from the Bolgatanga Municipal Health Directorate revealed that liver cirrhosis was among the first three of the top ten diseases causing mortality from 2013 to 2015 [9]. It is, however, not clear what might be contributing to this trend, but hepatitis B is highly implicated due to the high seroprevalence of HBsAg (12.64%), among blood donors at the Bolgatanga Regional Hospital [10]. By their social and economic activities, market women are believed to be vulnerable to the HBV infection; however, there are limited studies on market women with regard to HBV prevalence, knowledge, and attitudes. This study, therefore, sought to determine the seroprevalence, knowledge, and attitudes of market women towards HBV infection in the Bolgatanga Municipality of the Upper East Region of Ghana.

## 2. Materials and Methods

### 2.1. Study Design and Setting

A descriptive cross-sectional study was conducted involving market women in the Bolgatanga Municipality from October 2017 to March 2018. A survey with a quantitative data collection procedure was employed.

**Figure 1:**
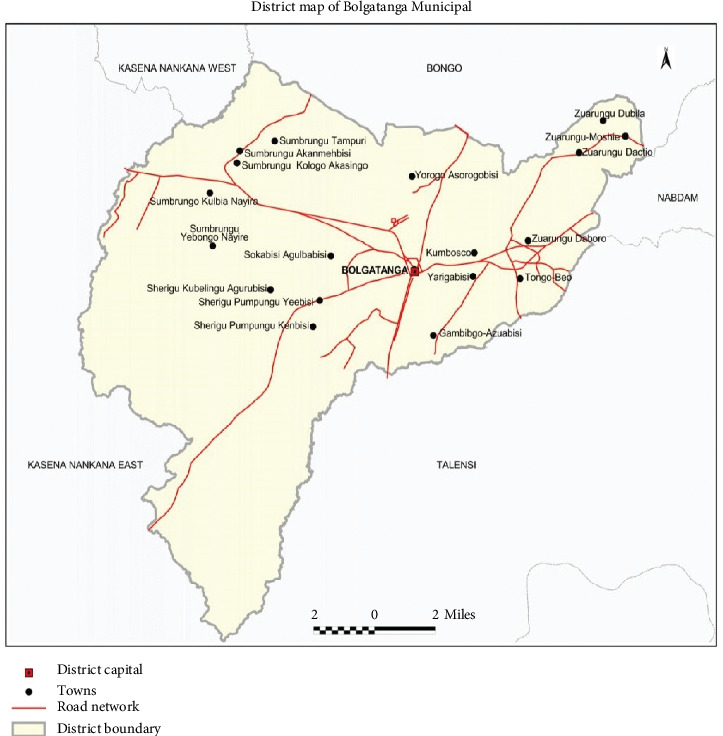
Map of Bolgatanga Municipal (source: GSS [11]).

The study was conducted in the Bolgatanga Municipality, which serves as the regional capital of the Upper East Region of Ghana, and has a total population of 131,550 [11]. The municipality has a total land area of 729 sq. km and is bordered to the north by the Bongo district, south and east by Talensi and Nabdam districts, respectively, and Kassena Nankana district to the west (Figure 1).

It is a municipality served by 34 health facilities; 1 regional hospital, 7 clinics, 6 health centres, and 20 CHPS compounds [12]. Licensed chemical shops and herbal practitioners, especially in the rural communities, augment these facilities. The spread of health facilities in the municipality is almost uniform with all communities within reasonable distances to health facilities. The regional hospital located, however, in the Bolgatanga Township in case of referrals is many kilometers from most of these communities and this has implications on the health of the people especially pregnant women among others [12].

### 2.2. Sampling of Study Participants and Sample Size Determination

A purposive sampling method was employed in the selection of the markets in the municipality. This technique was employed to sample out the market places in the municipality. After selecting the market women group of interest to the study objective, study subjects were then selected by a simple random approach.

Cochran formula [13] for infinite populations was used for determining the sample size of the study participants:

(1) $$\begin{array}{c} \text{sample size}\left(N_{\text{o}}\right)=\frac{z^{2}pq}{e^{2}}, \end{array}$$

where *p* = 0.5, *q* = 1 − 0.5 = 0.5, *z* is the confidence interval which is 1.96, *e* is the margin of error which is 0.05, *N*_o_ is the sample size, and *p* is the population at 50%, i.e., 0.5:

(2) $$\begin{array}{c} N_{\text{o}}=\frac{1.96\;\times 1.96\;\left(0.5\left(\;1-0.5\right)\right)}{0.05\times 0.05},\\ N_{\text{o}}=\frac{3.8416\left(0.25\right)}{0.0025},\\ N_{\text{o}}=3.8416\left(100\right),\\ \text{sample size }\left(N_{\text{o}}\right)=384. \end{array}$$

An attrition rate of 5% was added to the total sample size to cater for nonresponses. Five percent (5%) of the total sample size (384), which is 20 rounded to the nearest decimal, finally gave a total sample size of 404 respondents.

### 2.3. Data Collection Methods/Tools

The study involved the use of a pretested semistructured questionnaire to assess the knowledge and attitudes of market women towards the HBV infection. Enumerators were given a one-day training prior to the questionnaire administration and the screening of HBV. HBV status was determined by the use of HBsAg Rapid Diagnostic Test strips (RDTs) (Perfect, United States of America, Batch/Lot number 20160728). Briefly, the fingertip of the subject was cleaned with an alcohol swab and a lancet was used to prick the finger. Three drops of buffer solution were added to the strip on which the drop of whole blood obtained after pricking the fingertip of the study subject was placed. The result was read 3–5 minutes later and interpreted following the manufacturer's instructions.

### 2.4. Inclusion and Exclusion Criteria

Market women were selected based on the groups they belonged to (for example, yam sellers and tomato sellers) and were contacted through their leaders. The list of groups and what they sell were obtained from the municipal assembly. The aims and objectives of the study were explained to the leaders who in turn shared with their members. There was close monitoring of the shared information to ensure that the rationale of the study was clear to the respondents. Any consenting woman who had not been vaccinated against the Hep B virus was eligible to be recruited for the study. From each of the groups, the study subjects were randomly selected using the lottery method to arrive at the 404 study respondents. Respondents were interviewed using the pretested questionnaire. The other groups excluded from this study were smaller market centres within the municipality who had not registered with the assembly as well as those who also did not consent.

### 2.5. Data Analysis

All questionnaires were examined for completeness, consistency, and clarity as part of data management. The data were coded, entered, and cleaned before analyses using SPSS version 21.0. Descriptive analyses were run to determine the prevalence of HBV in the study population and its distribution categorized by independent variables. Also, the chi-square test was computed at a 95% confidence interval and 0.05% margin of error. To measure the levels of knowledge and attitude towards the infection, a scale of 0–14 (for knowledge scoring) and 0–4 (for attitude scoring) was designed based on the number of questions under each variable. Composite scores were obtained from individual scorings and put together or categorised to ascertain the percentage of the population with low, moderate, or high knowledge and poor or good attitude towards the HBV infection. Analyses were considered statistically significant at *p* < 0.05.

### 2.6. Ethical Considerations

Ethical approval was obtained from the Navrongo Health Research Centre Institutional Review Board (Ethics Approval ID: NHRCIRB283). An introductory letter was also obtained from the University for Development Studies—Department of Public Health, School of Allied Health Sciences (SAHS). Furthermore, permission to carry out the study in the municipality was granted by the Municipal Assembly and Health Directorate in the Upper East region. Informed consent was sought from the participants before the questionnaire was administered to them. They were assured of confidentiality and liberty to withdraw from the study if they felt uncomfortable.

### 2.7. Validity and Reliability

For the purpose of validity, the content of the questionnaire reflected the objectives of the study. Appropriate literature review was also ensured. Reliability was ensured through the explanation of terms and concepts in clear and understanding form, collection of right information, and usage of systematic methodology.

### 2.8. Study Limitation

Nurses who were recruited as part of the study to take the blood samples were not natives of the region, and thus, there was a language barrier between them and the study participants. This was addressed by getting the enumerators who were fluent in the local dialect explain to them before their blood samples were taken for testing. The use of a purposive sampling technique may be subjected to selection bias and can limit the generalization of the findings. Nevertheless, purposive selection has an advantage where it is easier to make generalisations about the sample used in the study as all participants are more likely to have the characteristics the study is interested in.

## 3. Results

### 3.1. Sociodemographic Characteristics of Market Women and Prevalence of Hepatitis B

A total of 404 women were assessed during the study period. All the respondents were identified from the Bolgatanga municipal market. The mean and modal ages were 37.7 years and 28.0 years, respectively. With regard to the educational status of the study participants, it was observed that 37.4% had nonformal education while 22%, 11.6%, and 5.4% had primary, junior high, and senior high education, respectively (Table 1). Meanwhile, 23.5% had neither some form of education nor training. At the time of the study, 68.6% were married, with 13.9% divorced and 13.6% single, while the remaining 4% were widowed. The results of the survey also showed that about 58% were Christians while 30.9% were Muslims and the rest traditionalists. Among the 404 study participants who were tested for the hepatitis B surface antigen (HBsAg), 63 were reactive. Prevalence of HBV infection among the market women was therefore 15.6%.

### 3.2. Relationship between Sociodemographic Characteristics and Hepatitis Status

The relationship between the sociodemographic characteristics and respondents' HBV status was determined using chi-square analysis. It was observed that there were no statistical correlations between the reactive and nonreactive individuals for HBV with respect to age (*p*=0.686), religion (*p*=0.102), and marital status (*p*=0.524), as well as educational status (*p*=0.056) (Table 2).

### 3.3. Awareness and Knowledge Levels of Hepatitis B Viral Infection

In the study, all participants were asked whether they had heard about HBV before and 57.2% answered in the affirmative. Most of the study participants (82.7%) answered “no” to “*if a healthy person can be vaccinated for Hepatitis B*” (Table 3). Majority of the study participants (75.9%) answered “no” to whether HBV infection could be inherited from parents. More than half of the study participants (66.3%) were unable to tell whether HBV infection could be transmitted through sex. On further analysis on the transmission mode, over 66.5% indicated that HBV infection could be transmitted through sharing of sharp objects, 75.2% also were of the view that HBV could be transmitted through delivery. However, 83.5% stated that HBV infection could not be transmitted through sharing of bowls and spoons, while 72.5% and 73.8% of the respondents answered “no” to whether HBV infection could be transmitted through kissing and sharing of toothbrushes, respectively (Table 3).

The findings also revealed that 6.9% of the study participants indicated HBV infection could be transmitted through holding hands. It was also observed that 59.4% were not aware HBV infection could cause damage to the liver, while 60.4% did not know whether HBV infection could be spread by an infected person (Table 3). The study further revealed that 67.0% of the participants did not know there was treatment for HBV infection; 19.7% were of the opinion that eating healthily and exercising could prevent a person from contracting the infection.

### 3.4. Rating Respondents Level of Knowledge on Hepatitis B Infection

Respondents were rated according to their responses to the questions pertaining to knowledge (Table 4), which were scored as “1” for a correct response and “0” for a wrong response and subsequently summed up for each respondent (composite scoring). Majority (61.6%) of the respondents scored between 0 and 4, indicating low knowledge, while 27.2% had scores within the range of 5–9 signifying moderate knowledge whilst 11.1% scored 10–14, indicating high knowledge.

### 3.5. Attitudes and Practices of Study Participants towards Hepatitis B Virus Infection

Respondents were asked some questions in relation to their attitudes towards the hepatitis B virus infection (Table 5). On assessing the attitudes of participants towards HBV infection, majority of them (48.5%) felt they did not need a vaccination currently. However, 92.1% of respondents said infected persons should not be isolated. Majority of the study participants (62.9%) felt a healthy person should not go for vaccination. Majority (87.8%) of the study subjects also declared that they would not have sex with their infected partners.

### 3.6. Rating of Respondents Attitude towards HBV

Most of the respondents (90.8%) scored from 0 to 2, indicating a bad attitude towards the condition, while 9.2% scored 3-4, thus having a good attitude (Table 6).

### 3.7. Relationship between Attitude and Hepatitis B Status

Three of the attitudinal questions did not have any significant relationship with the participants' hepatitis B status while the other one on “*whether infected people be isolated*” was significantly related to the hepatitis B status (Table 7).

## 4. Discussion

### 4.1. Prevalence of Hepatitis B Virus Infection among Respondents

The prevalence of HBV infection (using HBsAg) among market women screened in the study was 15.6%. This level of prevalence may compare with those reported among blood donors and pregnant women within the 20–60 age bracket [6, 14–16]. These studies reported varied results on HBV prevalence ranging from 10.8% to 16.4% in similar settings. The discrepancies in prevalence rates realized in the reported studies and the current study could be as a result of the different geographical locations, age, occupation, and lifestyle. It is speculated that higher prevalence observed in some of the studies could be as a result of the particular geographical locations where the studies were conducted, but this remains to be explored in further studies. Currently, there are more than 350 million people living with hepatitis B, and the consequence of this is approximately 600,000 HBV-related deaths every year around the world, where the cause is primarily liver cirrhosis or liver cancer [1]. Every year, there are over 4 million acute clinical cases of HBV, and about 25% of carriers die from chronic active hepatitis, cirrhosis, or primary liver cancer [17]. This further confirms that Ghana is grouped among the high endemic category according to World Health Organization categorization.

### 4.2. Awareness and Knowledge of Hepatitis B

Findings from the study have revealed that about 57.2% of the respondents were aware of HBV infection. Nevertheless, majority of the study subjects had poor knowledge of the HBV and its infection due to the fact that 61.6% of respondents scored within the ranges of 0–4 out of 14 questions on knowledge correctly. The seemingly high unawareness level in this study may be attributed to the awareness outcomes created among blood donors and pregnant women who have shown high prevalence rates in the region. The findings, however, contradict that of Taylor et al. [18], who investigated the knowledge and awareness of hepatitis B among randomly selected Vietnamese adults living in the United States and found that their knowledge level of the infection was generally good. This variation could be due to the fact that respondents in the latter study [18] lived in a more advanced country where conditions of public health significance are treated with utmost importance which could have contributed to their knowledge of the disease. The current study, however, is in concordance with a study by Ali Abdulai and colleagues [19], who observed a low level of knowledge and awareness of HBV among pregnant women attending antenatal clinics in two facilities in the Kintampo North Municipality. Other studies have shown that factors associated with good knowledge were being in the 35- to 44-year age group, ethnicity, high educational attainment, and high family income [20]. Rajamoorthy et al. thus observed that being older and having high educational attainment were determinants of having good awareness towards HBV. The study by Rajamoorthy et al. [20] emphasized the need for education to increase awareness on HBV infection. In their study, having good knowledge enabled participants to be 2.5 times more likely to have good awareness.

Hepatitis B, mostly known as the “*secret killer*,” is a major threat to health globally; however, it is yet to catch the attention of most health institutions, policy makers, the general public, and decision makers in Ghana. It is imperative that people be made aware and knowledgeable about the disease which will help them take precautions to prevent the infection. As the saying goes “*Prevention is better than cure*,” thus, prevention is one of the best ways to safeguard a populations' health.

### 4.3. Attitudes of Respondents towards Hepatitis B Virus Infection

It was evident from the current study that respondents' attitude towards HBV infection was generally poor. Despite the low knowledge levels about the HBV infection, most of the respondents (62.9%) saw the need for healthy individuals to be protected via vaccination. This is because respondents seem to be familiar with the benefits of vaccination. This is similar to the study in Kintampo [21] as inhabitants in Bolgatanga and Kintampo do not have different characteristics or behaviors.

Due to fear of the infection, majority of the respondents (87.8%) would not have sex with their infected partners. In addition, a higher proportion (92.1%) thinks that an infected individual should not be isolated to prevent spreading among the healthier ones. It is not clear if the low knowledge level about the HBV infection might be a contributor for 48.5% of participants to indicate no need of vaccination. It is tempting to speculate that lower literacy levels among the market women may be implicated as attitude is reported to have an association with knowledge [22].

Most of the respondents probably did not have time to go for screening or vaccination. This is due to the fact that most of the times they are doing business at the market and ignoring their health care. Most of the respondents who were reactive to HBV test had no idea whether they needed to take the vaccine or not, which goes to buttress the low knowledge and poor attitude among the respondents. Additionally, 54.5% of nonreactive respondents had no idea about whether or not it was appropriate to get vaccinated. Hepatitis B virus infection is largely preventable by a vaccine which is 95% effective against the disease and its chronic consequences. Transmission of the infection is rare among immunized persons.

Upon interaction with the market women in the Bolgatanga Municipality, it was evident that many of them have little or average knowledge with regard to the functions their liver play in the maintenance of their health. Even after testing reactive to the HBV infection, they thought they were healthy since they had not really experienced any significant signs and symptoms of the disease, and for that matter, they saw no need to waste their time getting screened or vaccinated. This buttresses the impact low knowledge level and poor attitude towards HBV infection have on the disease. Complacency among these women may also be attributed to poor attitude, which prevented them from enquiring about the HBV infection, as it is culturally perceived that most people from Northern Ghana are physically active and strong and therefore cannot succumb to most sicknesses including hepatitis B [21].

It was observed from the study that those who were between the ages of 30 and 39 years had the highest HBV positivity. Anderson et al. [23] reported high prevalence of HIV-1 among 24- to 54-year-olds in urban areas of Tanzania and this was probably attributed to high sexual activity. Even though our study did not explore the role of sexual activity, it is one of the means by which HBV is contracted. Thus, it is tempting to speculate that the highest HBV positivity among 30-to 39-year-olds within the Bolgatanga Municipality could be attributed to high sexual activity among them. Furthermore, the anecdotal report suggests that most market women patronize the local “manicurist” who use unsterilized tools as well as sharing needles, blades, and other sharp objects which make them prone to the HBV infection. Africans by nature have a communal lifestyle of living which brings them closely together [24] which could be why most of the respondents responded negatively to the isolation of HBV-infected individuals from their communities.

## 5. Conclusions

The HBV prevalence among the study respondents has been shown to be high of 15.6%, and also, most of them had merely heard of the hepatitis infection but had no idea about what it entails, hence a relatively low knowledge level among the market women about the infection in the Bolgatanga Municipality. Even though the majority of the respondents had some form of education, there was no significant association between their educational levels and hepatitis B status. There was generally low level of knowledge and poor attitude towards the infection among the study participants.

## 6. Recommendation

Based on the findings in the study, it is recommended that the Ghana Health Service and Ministry of Health carry out a free compulsory market to market vaccination program, while Municipal Assembly and District Health Directorate should organize and implement educational programmes from time to time to sensitize market women and the entire municipality about the HBV infection. Further studies are recommended to include other vulnerable workers and men to confirm the high HBV prevalence.

## Figures and Tables

**Table 1:** Sociodemographic characteristics of market women.

Variable	Frequency	Percentages (%)
Age (years)		
<30	103	25.5
30–34	67	16.6
35–39	67	16.6
40–44	56	13.9
45–49	42	10.4
50+	69	17.1
**Total**	**404**	**100.0**
Educational status		
Nonformal	151	37.4
Primary	89	22.0
JHS	47	11.6
SHS	22	5.4
None	95	23.5
**Total**	**404**	**100.0**
Religion		
Christianity	234	57.9
Islam	125	30.9
Traditional	45	11.1
**Total**	**404**	**100.0**
Marital status		
Married	277	68.6
Single	55	13.6
Divorce	16	4.0
Widow	56	13.9
**Total**	**404**	**100.0**

Source: Field Survey, 2017.

**Table 2:** Relationship between sociodemographic characteristics and hepatitis B status.

Variable	Frequency		*p* value
	Reactive to HBV	Nonreactive to HBV	
Age (years)			
<30	14	89	0.686
30–34	14	53	
35–39	12	55	
40–44	6	50	
45–49	6	36	
50+	11	58	
**Total**	**63**	**341**	**404**
Level of education			
Nonformal	26	125	0.056
Primary	8	81	
JHS	9	38	
SHS	3	19	
None	17	78	
**Total**	**63**	**341**	**404**
Religion			
Christianity	29	205	0.102
Islam	26	99	
Traditional	8	37	
**Total**	**63**	**341**	**404**
Marital status			
Married	40	237	0.524
Single	11	44	
Divorce	4	12	
Window	8	48	
**Total**	**63**	**341**	**404**

Source: Field Survey, 2017.

**Table 3:** Awareness and knowledge levels of hepatitis B viral infection.

Question	Yes	No
Heard of hepatitis B infection	231 (57.2%)	173 (42.8%)
Is hepatitis B inherited?	98 (24.3%)	306 (75.9%)
Can healthy person be vaccinated for HBV?	70 (17.3%)	334 (82.7%)
Can infected person spread HBV?	159 (39.4%)	244 (60.4%)
Are jaundice, fatigue, and nausea signs of HBV?	64 (15.7%)	340 (83.5%)
Is hepatitis B transmitted through sex?	136 (33.7%)	268 (66.3%)
Is hepatitis B transmitted through sharing sharp objects?	135 (33.5%)	268 (66.5%)
Is hepatitis B transmitted through delivery?	100 (24.8%)	304 (75.2%)
Is hepatitis B transmitted through sharing bowls and spoons?	68 (16.8%)	336 (83.2%)
Is hepatitis B transmitted through kissing?	111 (27.5%)	293 (72.5%)
Is hepatitis B transmitted through toothbrush sharing?	106 (26.2%)	298 (73.8%)
Is hepatitis B transmitted through holding hands?	28 (6.9%)	376 (93.1%)
Is there treatment for hepatitis B?	133 (32.9%)	271 (67.0%)
Does exercising and eating healthy prevent hepatitis B?	79 (19.7%)	325 (80.4%)
Can HBV cause liver infection?	164 (40.6%)	240 (59.4%)

Source: Field Survey, 2017.

**Table 4:** Rating of respondents' knowledge level on HBV infection.

Variable (knowledge)	Scale	Frequency	Percentage (%)
Low	0–4	249	61.6
Moderate	5–9	110	27.2
High	10–14	45	11.1

Total		404	100

Source: Field Survey, 2017.

**Table 5:** Attitudes/practices of people towards hepatitis B virus infection.

Question	Yes	No
Do you feel a healthy person should get vaccinated?	150 (37.1%)	254 (62.9%)
Would you have sex with an infected partner?	49 (12.2%)	355 (87.8%)
Should infected people be isolated?	32 (7.9%)	372 (92.1%)
Do you feel you need a vaccination currently?	208 (51.5%)	196 (48.5%)

Source: Field Survey, 2017.

**Table 6:** Rating of respondents' attitude towards HBV infection.

Variable (attitude)	Scale	Frequency	Percentage (%)
Bad	0–2	367	90.8
Good	3–4	37	9.2
Total		404	100

Source: Field Survey, 2017.

**Table 7:** Relationship between attitude and hepatitis B status.

Question	Reactive	Nonreactive	Total	*p* value
Should a healthy person go for vaccination?				
Yes	21	129	150	0.493
No	3	26	29	
Don't know	39	186	225	
Total	63	341	404	
Would you have sex with an infected partner?				
Yes	9	40	49	0.373
No	53	292	345	
Don't know	1	9	9	
Total	63	341	404	
Should infected people be isolated?				
Yes	1	31	32	**0.049**
No	51	230	281	
Don't know	11	80	91	
Total	63	341	404	
Do you feel you need a vaccination currently?				
Yes	24	184	208	0.058
No	11	38	49	
Don't know	28	119	147	
Total	63	341	404	

Source: Field Survey, 2017.

## Data Availability

The SPSS data used to support the findings of this study are available from the corresponding author upon request.
